# Translational study reveals a two-faced role of RBM3 in pancreatic cancer and suggests its potential value as a biomarker for improved patient stratification

**DOI:** 10.18632/oncotarget.23486

**Published:** 2017-12-15

**Authors:** Emelie Karnevi, Liv Ben Dror, Adil Mardinoglu, Jacob Elebro, Margareta Heby, Sven-Erik Olofsson, Björn Nodin, Jakob Eberhard, William Gallagher, Mathias Uhlén, Karin Jirström

**Affiliations:** ^1^ Division of Oncology and Pathology, Department of Clinical Sciences, Lund University, Skåne University Hospital, Lund, Sweden; ^2^ Science for Life Laboratory, KTH, Royal Institute of Technology, Stockholm, Sweden; ^3^ UCD School of Biomolecular and Biomedical Science, UCD Conway Institute, 31 University College Dublin, Dublin, Ireland

**Keywords:** RBM3, pancreatic cancer, periampullary cancer, prognosis, prediction

## Abstract

Periampullary adenocarcinoma, including pancreatic cancer, is a heterogeneous group of tumors with dismal prognosis, partially due to lack of reliable targetable and predictive biomarkers. RNA-binding motif protein 3 (RBM3) has previously been shown to be an independent prognostic and predictive biomarker in several types of cancer. Herein, we examined the prognostic value of RBM3 in periampullary adenocarcinoma, as well as the effects following RBM3 suppression in pancreatic cancer cells *in vitro*. RBM3 mRNA levels were examined in 176 pancreatic cancer patients from The Cancer Genome Atlas. Immunohistochemical expression of RBM3 was analyzed in tissue microarrays with primary tumors and paired lymph node metastases from 175 consecutive patients with resected periampullary adenocarcinoma. Pancreatic cancer cells were transfected with anti-RBM3 siRNA *in vitro* and the influence on cell viability following chemotherapy, transwell migration and invasion was assessed. The results demonstrated that high mRNA-levels of RBM3 were significantly associated with a reduced overall survival (*p* = 0.026). RBM3 protein expression was significantly higher in lymph node metastases than in primary tumors (*p* = 0.005). High RBM3 protein expression was an independent predictive factor for the effect of adjuvant chemotherapy and an independent negative prognostic factor in untreated patients (*p* for interactio*n =* 0.003). After siRNA suppression of RBM3 *in vitro*, pancreatic cancer cells displayed reduced migration and invasion compared to control, as well as a significantly increased resistance to chemotherapy. In conclusion, the strong indication of a positive response predictive effect of RBM3 expression in pancreatic cancer may be highly relevant in the clinical setting and merits further validation.

## INTRODUCTION

Periampullary adenocarcinomas can be found in the head of the pancreas in the region of the ampulla of Vater and comprise ampullary, pancreatic, bile duct and perivaterian duodenal cancer - a group of heterogeneous tumors all having a rather dismal prognosis [1, 2]. For resected periampullary tumors, further separation by histological type of differentiation into intestinal type (I-type) or pancreatobiliary types (PB-type) has been shown to provide important prognostic information, whereby the latter is associated with the poorest clinical outcome [3]. Advanced pancreatic and periampullary cancer is very complex to treat; whenever surgical resection is possible, adjuvant chemotherapy often follows and most commonly includes gemcitabine or 5-FU, a management shown to improve survival [1, 4]. However, since only 15–20% of patients are eligible for surgery, the 5-year overall survival for all patients with pancreatic adenocarcinoma is less than 5%, contributing to almost identical incidence and death rates. For periampullary cancer patients, excluding pancreatic cancer, the survival is somewhat better with a 5-year overall survival of approximately 20% [5]. The disease is number four among leading causes of cancer death in the United States [2, 6]. Thus, there is an immense need for better molecular tools to assist in an improved prognostic and treatment predictive stratification of patients with these types of cancers.

High expression of the RNA-binding motif protein 3 (RBM3) has been demonstrated to be an independent prognostic and predictive biomarker in several types of solid tumors such as malignant melanoma, breast, ovarian, prostate, bladder, colorectal, esophageal, gastric, and non-seminomatous testicular cancer [7–15]. In addition, siRNA-mediated down-regulation of RBM3 in epithelial ovarian cancer cells has been demonstrated to confer a reduced sensitivity to platinum-based chemotherapy [8]. A possible link between RBM3 expression and DNA integrity and repair in ovarian cancer has been suggested [16], and RBM3 has also been demonstrated to attenuate stem cell like properties of prostate cancer cells [17]. Further insight into the mechanistic basis underlying the role of RBM3 in tumor progression and chemotherapy sensitivity is however needed. To our best knowledge, the prognostic significance of RBM3 in pancreatic and periampullary cancer has not yet been described.

The aim of the present study was therefore to examine the prognostic and potential predictive significance of immunohistochemical expression of RBM3 in primary tumors and a subset of paired lymph node metastases from a consecutive cohort of patients with periampullary adenocarcinoma. The prognostic value of RBM3 was also validated at the gene expression level in 176 tumors in The Cancer Genome Atlas (TCGA). In addition, the effects of siRNA-mediated knockdown of RBM3 on chemotherapy response and tumor cell migration and invasion were investigated *in vitro*.

## RESULTS

### RBM3 expression in primary tumors and lymph node metastases

**Figure 1:**
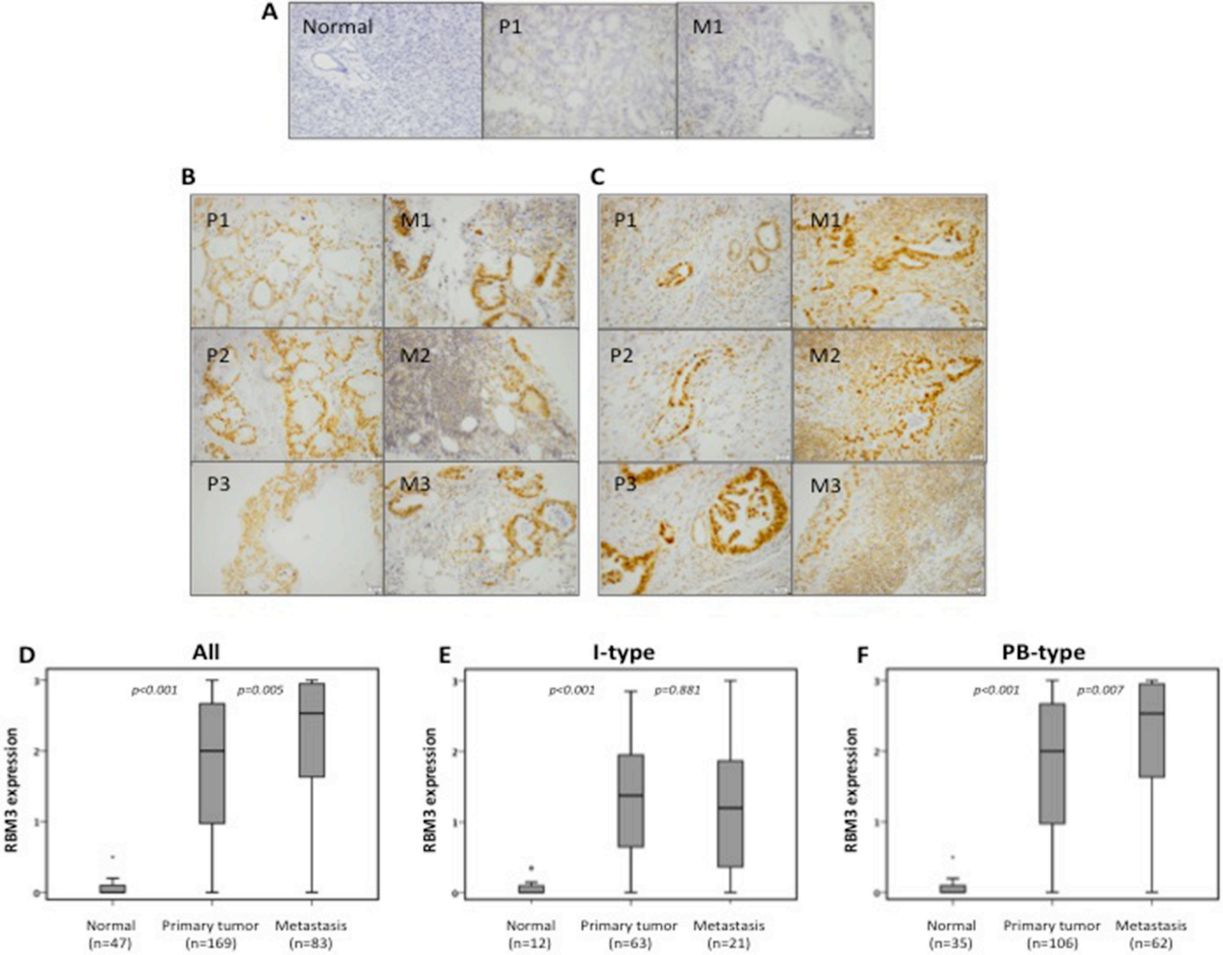
RBM3 expression in primary tumors and lymph node metastases Immunohistochemical images of (**A**) negative RBM3 expression, (**B**) intermediate RBM3 expression and (**C**) strong RBM3 expression. A) Negative staining in normal pancreatic tissue and paired intestinal type primary tumour and metastasis. B-C) Pictures represent three paired primary tumors and metastasis from intestinal type tumors (B) and pancreatobiliary-type tumors (C). Box plots visualizing the distribution of RBM3 expression in (**D**) the entire cohort, (**E**) intestinal type tumors and (**F**) pancreatobiliary-type tumors. The images were taken at 20X magnification using cellSens dimension software. Scale bar represents 20 μm.

RBM3 expression could be assessed in 171/175 (97.7%) of primary tumors and in 83/105 (79.0%) of sampled lymph node metastases. In normal pancreatic tissue, successful staining of RBM3 was achieved in 47/50 (94.0%) samples. Sample immunohistochemical images are shown in Figure 1A–1C. In line with previous studies, RBM3 was mainly differentially expressed in the nucleus [7–13], and no significant associations were found between cytoplasmic RBM3 expression and survival or any clinicopathological parameters (data not shown) and the results hereafter will only refer to the nuclear RBM3 expression. In the entire cohort, RBM3 expression was significantly higher in primary tumors as compared with normal pancreatic tissue (*p* < 0.001, Figure 1D) and significantly higher in lymph node metastases than in primary tumors (*p* = 0.005, Figure 1D). Similar results were seen in the pancreatobiliary (PB) type tumors (Figure 1F) whereas in the intestinal (I) type tumors, RBM3 expression did not differ significantly between primary tumors and lymph node metastases (Figure 1E).

### Association of RBM3 expression in primary tumors with clinicopathological parameters

The associations of RBM3 expression with clinicopathological factors in the full cohort and stratified according to histological subtype are shown in Table 1. In the entire cohort, significant associations were found between RBM3 expression and growth into peripancreatic fat (*p* = 0.006) and in perineural invasion (*p* = 0.023). When comparing the two morphological subtypes, RBM3 expression was significantly higher in PB-type tumors than I-type tumors (*p* = 0.002), and as shown in Table 1, the highest expression was seen in pancreatic cancer. For I-type tumors, a significant inverse association was found between RBM3 expression and tumor size (*p* = 0.030), and in PB-type tumors, RBM3 expression was significantly associated with the presence of lymph node metastases (*p* = 0.015), larger tumor size (*p* = 0.031), and with growth in peripancreatic fat (*p* = 0.027).

**Table 1:** Associations of RBM3 expression in primary tumors with clinicopathological parameters

	All			Intestinal type			Pancreatobiliary type		
	*n*	median (range)	P	*n*	median (range)	P	*n*	median (range)	P
**Age**									
Q1 (38–61)	39	1.81 (0.13–3.00)	0.799	18	1.82 (0.27–2.85)	0.539	21	1.81 (0.13–3.00)	0.413
Q2 (62–67)	43	1.70 (0.03–3.00)		13	1.10 (0.03–2.70)		30	2.03 (0.05–3.00)	
Q3 (68–72)	44	1.80 (0.00–3.00)		19	1.22 (0.00–2.67)		25	2.27 (0.00–3.00)	
Q4 (73–84)	43	1.52 (0.00–3.00)		13	1.33 (0.07–2.75)		30	1.61 (0.00–3.00)	
**Gender**									
Female	84	1.80 (0.00–3.00)	0.768	34	1.53 (0.03–2.75)	0.324	50	2.00 (0.00–3.00)	0.887
Male	85	1.60 (0.00–3.00)		29	1.30 (0.00–2.85)		56	2.00 (0.00–3.00)	
**Tumor origin**									
Duodenum	14	1.30 (0.00–2.85)	0.052	14	1.30 (0.00–2.85)	0.655		–	0.988
Papilla-ampulla I-type	49	1.43 (0.07–2.70)		49	1.43 (0.07–2.70)			–	
Papilla-ampulla PB-type	18	2.00 (0.60–3.00)			–		18	2.00 (0.60–3.00)	
Distal bile duct	45	2.00 (0.00–3.00)			–		45	2.00 (0.00–3.00)	
Pancreas	43	2.07 (0.00–3.00)			–		43	2.07 (0.00–3.00)	
**Tumor origin dichotomized**									
Intestinal	63	1.38 (0.00–2.85)	**0.002**		–			–	
Pancreatobiliary	106	2.00 (0.00–3.00)			–			–	
T–stage^*^									
T1	6	1.70 (0.92–2.55)	0.280	4	2.04 (1.47–2.55)	0.055	2	1.23 (0.92–1.53)	0.786
T2	21	1.90 (0.00–2.93)		11	1.63 (0.13–2.70)		10	2.25 (0.00–2.93)	
T3	103	1.83 (0.00–3.00)		25	1.43 (0.07–2.85)		78	2.00 (0.00–3.00)	
T4	39	1.37 (0.00–3.00)		23	1.00 (0.00–2.55)		16	2.00 (0.60–3.00)	
Lymph node metastasis^*^									
0	63	1.33 (0.00–3.00)	0.086	33	1.60 (0.03–2.67)	0.160	30	1.13 (0.00–3.00)	**0.015**
1–3	63	1.80 (0.00–3.00)		19	1.40 (0.00–2.85)		44	1.91 (0.00–3.00)	
4 or more	43	2.00 (0.05–3.00)		11	0.37 (0.10–2.05)		32	2.28 (0.05–3.00)	
**Tumor grade**									
Well	11	1.47 (0.07–3.00)	0.249	5	1.47 (0.07–2.22)	0.618	6	1.51 (0.92–3.00)	0.377
Moderate	58	1.57 (0.00–3.00)		26	1.22 (0.10–2.48)		32	1.95 (0.00–3.00)	
Poor	96	1.92 (0.00–3.00)		32	1.47 (0.00–2.85)		64	2.12 (0.00–3.00)	
**Tumor size**									
< = 20 mm	37	1.53 (0.07–2.90)	0.349	23	1.67 (0.07–2.70)	**0.030**	14	1.06 (0.10–2.90)	**0.031**
> 20 mm	132	1.80 (0.00–3.00)		40	1.12 (0.00–2.85)		92	2.03 (0.00–3.00)	
**Resection margins**									
R0	23	1.88 (0.13–3.00)	0.063	17	1.87 (0.13–2.75)	0.171	6	2.28 (0.92–3.00)	0.638
R1	92	1.92 (0.00–3.00)		14	1.65 (0.00–2.85)		78	2.03 (0.00–3.00)	
RX	54	1.36 (0.03–3.00)		32	1.08 (0.03–2.70)		22	1.85 (0.10–3.00)	
**Perineural growth**									
No	66	1.48 (0.00–3.00)	**0.023**	44	1.48 (0.03–2.75)	0.252	22	1.52 (0.00–3.00)	0.065
Yes	103	1.90 (0.00–3.00)		19	1.10 (0.00–2.85)		84	2.00 (0.05–3.00)	
**Lymphatic invasion**									
No	61	1.63 (0.00–3.00)	0.526	29	1.47 (0.00–2.75)	0.634	32	1.81 (0.00–3.00)	0.738
Yes	108	1.80 (0.00–3.00)		34	1.34 (0.07–2.85)		74	2.00 (0.00–3.00)	
**Vascular invasion**									
No	128	1.62 (0.00–3.00)	0.274	58	1.39 (0.00–2.85)	0.648	70	1.97 (0.00–3.00)	0.718
Yes	41	2.00 (0.00–3.00)		5	1.00 (0.10–2.55)		36	2.26 (0.00–3.00)	
**Growth in peripancreatic fat**									
No	63	1.40 (0.00–3.00)	**0.006**	41	1.43 (0.00–2.70)	0.751	22	1.27 (0.00–3.00)	**0.027**
Yes	106	1.92 (0.00–3.00)		22	1.23 (0.10–2.85)		84	2.08 (0.00–3.00)	

^*^Pathological staging of T and N.

### Prognostic value of RBM3 protein expression overall and in strata according to adjuvant treatment

**Figure 2:**
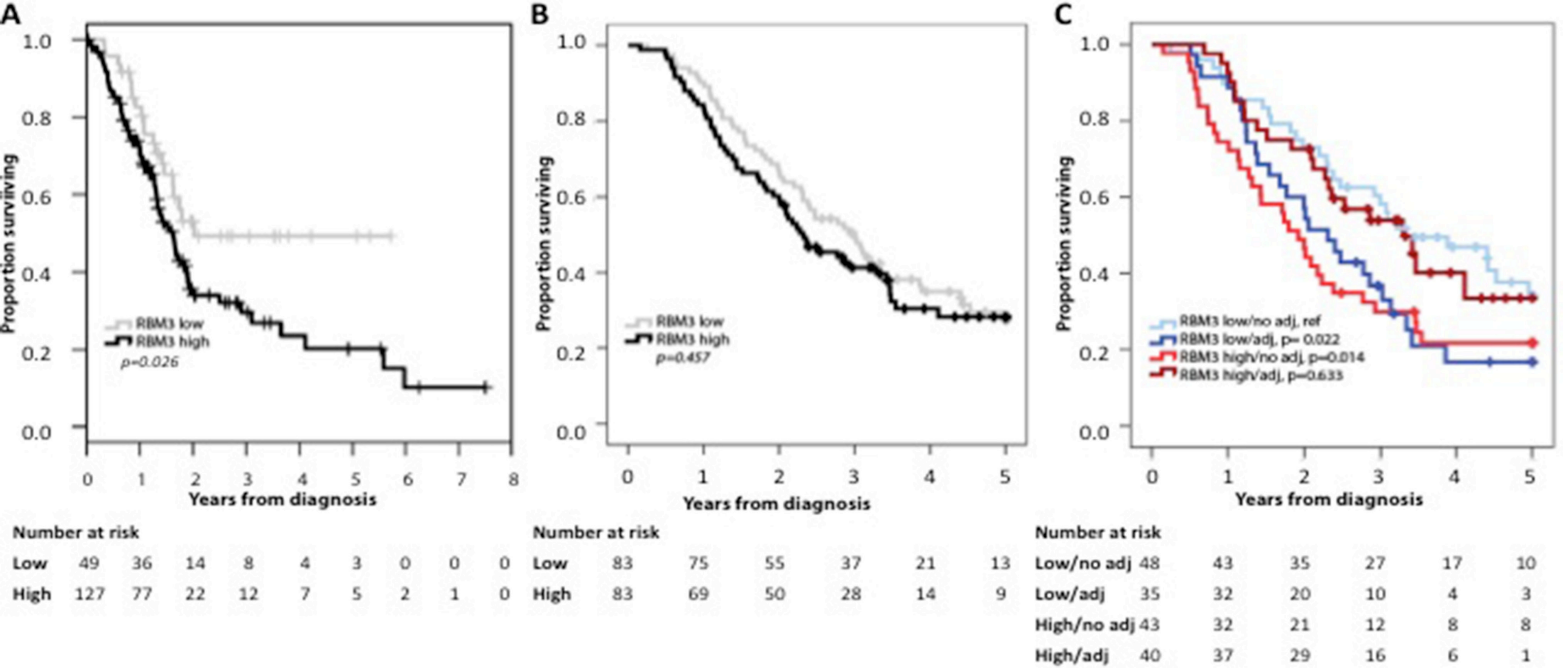
Prognostic value of RBM3 expression Kaplan-Meier analysis of 5-year overall survival in relation to (**A**) RBM3 mRNA expression in pancreatic cancer patients from the TCGA, (**B**) RBM3 protein expression in the periampullary cohort using the median cutoff value of the nuclear score, and (**C**) overall survival in strata according to RBM3 expression and any adjuvant chemotherapy, with low RBM3 expression/no adjuvant treatment as reference. Log rank *p*-value for high RBM3 expression /adjuvant treatment compared to high RBM3 expression/ no adjuvant treatment *p* = 0.035, and log rank *p*-value for high RBM3 expression /adjuvant treatment compared to low RBM3 expression /adjuvant treatment *p* = 0.070.

As demonstrated in Figure 2A, Kaplan Meier analysis demonstrated that high RBM3 mRNA levels were significantly associated with a shorter OS in the TCGA dataset. At the protein level, no prognostic value could be demonstrated for RBM3 expression in the full cohort regarding OS (Figure 2B), with similar results for RFS (graph not shown). The lack of association of RBM3 expression with OS and RFS in the entire cohort and according to histological subtype was confirmed in Cox regression analysis (Supplementary Tables 1 and 2, respectively). However, and notably, analysis in strata according to RBM3 expression and adjuvant treatment (Figure 2C) demonstrated that patients with low tumor-specific RBM3 expression who did not receive adjuvant treatment or patients with high tumor-specific RBM3 expression who did receive adjuvant treatment had the best survival, whereas patients with high tumor-specific RBM3 expression who did not receive adjuvant treatment or patients with low tumor-specific RBM3 expression who did receive adjuvant treatment had the poorest survival. As further shown in Table 2, high RBM3 expression was an independent adverse prognostic factor in untreated patients and an independent favorable prognostic factor in treated patients, with a significant treatment interaction for both OS (*p* = 0.003) and RFS (*p* = 0.009). Similar findings were observed in PB-type tumors, with a significant treatment interaction regarding both OS and RFS (*p* < 0.001 for both).

**Table 2:** Unadjusted and adjusted Cox proportional hazards analysis of the impact of RBM3 median protein expression on overall and recurrence free survival according to adjuvant treatment

	Death within 5 years			Recurrence		
	HR (95% CI)	*n* (events, %)	p†	HR (95% CI)	*n* (events, %)	p†
**All**						
*No adjuvant treatment*						
RBM3 low	1.00	48 (29, 60.4%)		1	48 (26, 54.2%)	
RBM3 high	1.85 (1.12–3.06)	43 (33, 76.7%)		2.00 (1.20–3.33)	43 (34, 79.1%)	
	2.04 (1.14–3.67)			2.12 (1.13–3.96)		
Any adjuvant treatment^*^			**0.003**			**0.009**
RBM3 low	1	35 (27, 77.1%)		1	35 (28, 80.0%)	
RBM3 high	0.60 (0.34–1.05)	40 (22, 55.0%)		0.76 (0.45–1.29)	40 (28, 70.0%)	
	0.24 (0.12–0.49)			0.31 (0.16–0.59)		
**Pancreatobiliary type**						
*No adjuvant treatment*						
RBM3 low	1	22 (14, 63.6%)		1	22 (13, 59.1%)	
RBM3 high	*2.49 (1.27–4.86)*	26 (24, 92.3%)		*2*.86 (1.45–5.*65)*	26 (26, 100%)	
	*4.25 (1.52–11.87)*			*2.13 (0.81–5.64)*		
Any adjuvant treatment^*^			**< 0.001**			**< 0.001**
RBM3 low	1	24 (22, 91.7%)		1	24 (22, 91.7%)	
RBM3 high	*0.45 (0.24–0.85)*	33 (21, 63.6%)		0.60 (0.33–1.06)	33 (26, 78.8%)	
	*0.41 (0.21–0.80)*			0.36 (0.19–0.69)		

Multivariable analysis adjusted for age, gender, tumor origin, tumor size, T-stage, N-stage, differentiation grade, involved margins, lymphatic growth, vascular growth, perineural growth and growth in peripancreatic fat.

†*p* value for term of interaction by Cox multivariable analysis including treatment, the binary covariate RBM3 expression, any vs no treatment and a term of interaction.

*The majority of patients received gemcitabine alone (*n* = 51 and *n* = 44, respectively) or in combination (*n* = 6 and *n* = 4, respectively).

In I-type tumors, the prognostic value of RBM3-expression did not differ by adjuvant treatment and there was no significant interaction between RBM3 expression and adjuvant treatment (data not shown).

### Suppression of RBM3 expression *in vitro* does not alter COX-2 and IL-8 levels

**Figure 3:**
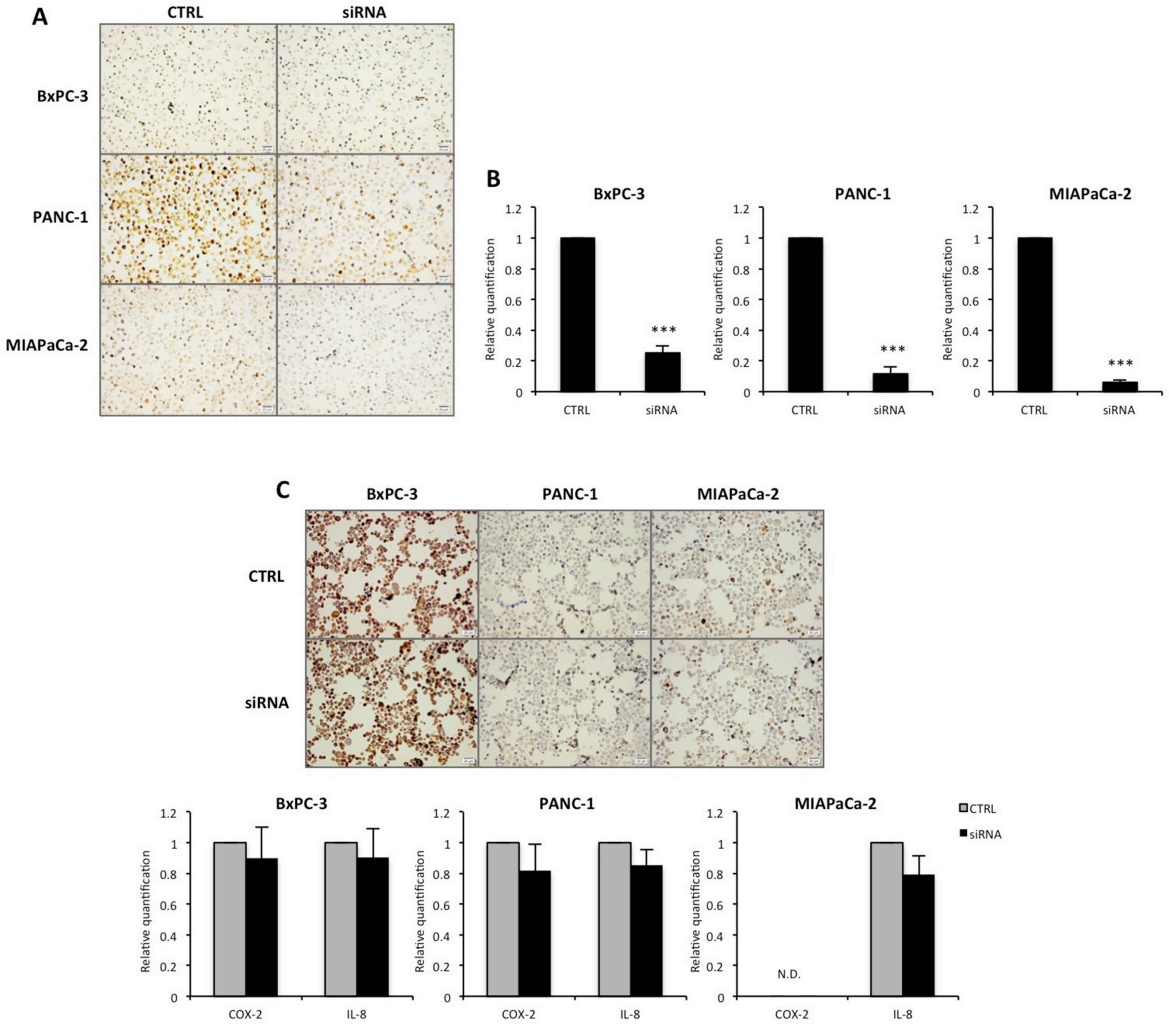
RBM3 mRNA and protein expression after transfection (**A**) Representative images of RBM3 protein expression in pancreatic cancer cell lines BxPC-3, PANC-1 and MIAPaCa-2 after transfection with siRNA against RBM3 or negative control (CTRL). (**B**) RBM3 relative mRNA expression levels in cancer cells after transfection with siRNA against RBM3 or siRNA negative control. (**C**) Representative images of COX-2 protein expression following transfection, as well as COX-2 and IL-8 relative mRNA expression levels in cancer cells after RBM3 suppression. Images and graphs represent one of at least three independent experiments. Representative images have been taken at 20X magnification with cellSens dimension software. Scale bar represents 20 μm. N.D. = not detected. ^***^p < 0.001

To investigate the role of RBM3 *in vitro*, pancreatic cancer cells were transfected with anti-RBM3 siRNA. As shown in Figure 3, the pancreatic cancer cells investigated display different levels of RBM3, where PANC-1 cells have high RBM3 expression, MIAPaCa-2 cells display moderate levels and BxPC-3 cells display low RBM3 expression (Figure 3A). Following transfection, the mRNA and protein levels of RBM3 were significantly reduced in all three cell lines, regardless of initial RBM3 expression (Figure 3A–3B). In addition, the mRNA levels of the two suggested downstream targets COX-2 and IL-8 [18] following transfection were evaluated. siRNA-mediated suppression of RBM3 did not alter the expression at neither mRNA nor protein level (Figure 3C). These findings were also in part supported by the baseline expression, where the expression of RBM3 did not correlate with neither COX-2 nor IL-8 expression in the cell lines investigated. In summary, RBM3 was shown to be successfully suppressed in this *in vitro* system, but did not affect the two suggested downstream targets COX-2 and IL-8.

### Effect of RBM3 suppression on cancer cell motility and chemotherapy sensitivity

**Figure 4:**
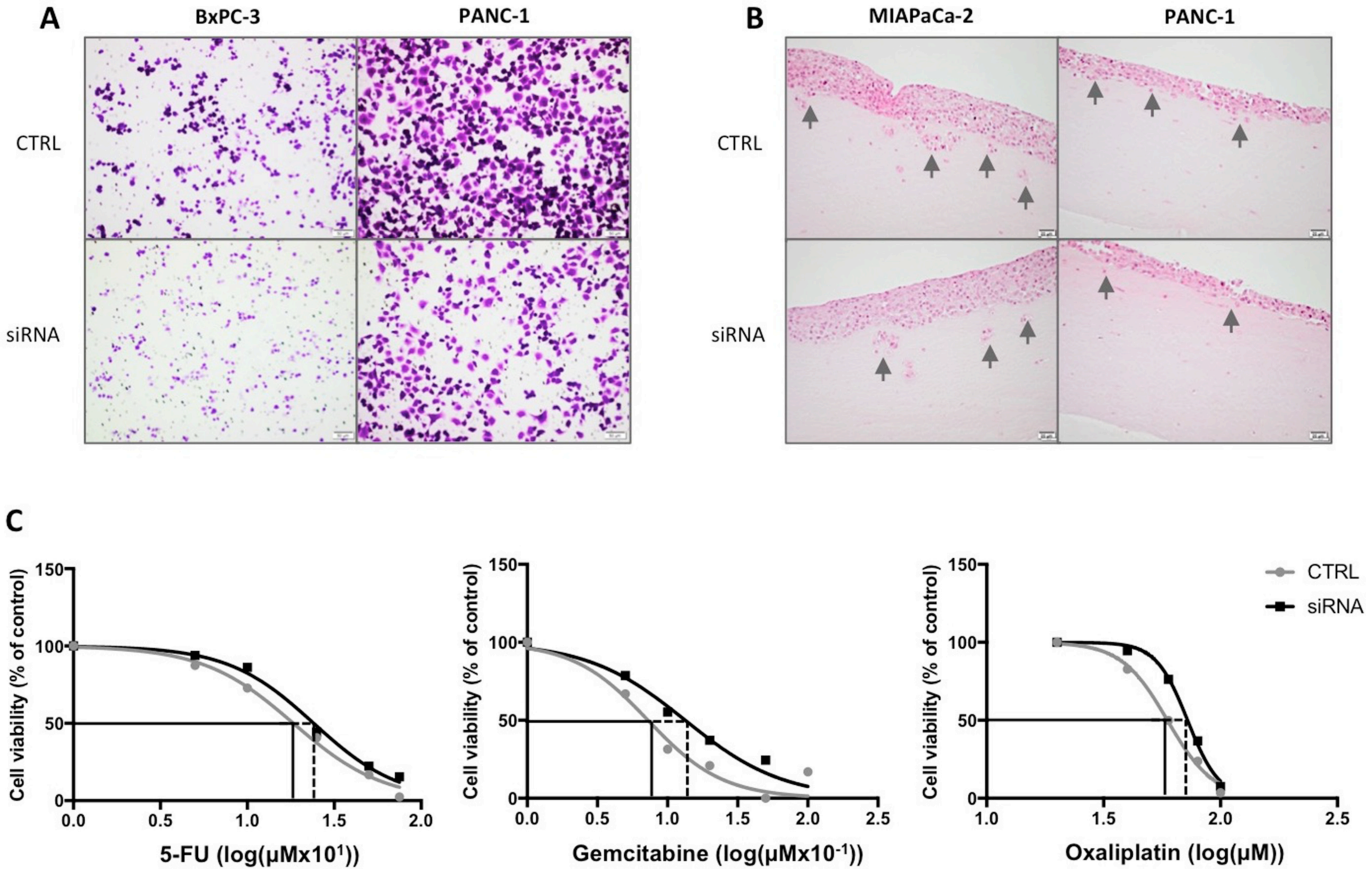
Influence by RBM3 suppression on cancer cell behavior (**A**) Representative images of transwell migration with BxPC-3 or PANC-1 cells after transfection with siRNA against RBM3 or control. (**B**) Representative images of organotypic gel sections stained with hematoxylin and eosin after 7 days incubation. All images were taken at 10X (A) or 20X (B) magnification using cellSens dimension software. Scale bar represents 20 (B) or 50 (A) μm. (**C**) Graphs represent cell viability after incubation with gemcitabine, oxaliplatin and 5-FU of MIAPaCa-2 cells with or without suppressed RBM3, relative to control (no treatment). Grey lines represent cells transfected with negative control siRNA, and black lines the RBM3 suppressed cells. Significant differences between control and siRNA were analyzed with non-linear regression.

The influence of RBM3 expression on pancreatic cancer cell migration and invasion was then investigated. Following siRNA transfection, pancreatic cancer cells were either seeded in transwell chambers or three-dimensional organotypic assay. BxPC-3 and PANC-1 cells demonstrated reduced migration after 14h incubation in transwell chambers (Figure 4A) and migrated MIAPaCa-2 cells were undetectable (Supplementary Figure 1). In a three-dimensional setting, the cells displayed decreased extent of invasion, most notably MIAPaCa-2, after 7 days incubation (Figure 4B). BxPC-3 cells are non-invasive in the three-dimensional model (Supplementary Figure 1).

Next, the influence of RBM3 on chemotherapy sensitivity was investigated. All three pancreatic cancer cell lines were incubated with gemcitabine following transfection. Of the three cell lines, only MIAPaCa-2 cells displayed decreased sensitivity when RBM3 was suppressed compared with control (*p* < 0.01; Figure 4C). PANC-1, with the highest baseline expression of RBM3, was shown to be resistant to gemcitabine and the low-expressing BxPC-3 cells displayed mixed response after transfection, rendering the two cell lines inappropriate as models (Supplementary Figure 1). Exposing MIAPaCa-2 cells to oxaliplatin and 5-FU displayed similar results to gemcitabine with reduced sensitivity in RBM3 suppressed cells compared with control (*p* < 0.05 and *p* < 0.001, respectively; Figure 4C).

In summary, in line with the clinical data, RBM3 expression is demonstrated to confer a more aggressive behavior by enhanced migration and invasion of pancreatic cancer cells, but also an increased sensitivity to chemotherapy *in vitro*.

## DISCUSSION

Periampullary adenocarcinomas, including pancreatic cancer, make up a heterogenos group with poor prognosis, even after surgical resection of the tumor. Among the challenges faced in the clinic are limited treatment options, advanced stage of the disease at diagnosis, lack of reliable biomarkers as well as ways of determining patients groups who would benefit from available treatments. This study is, to our best knowledge, the first to report on the expression and prognostic significance of RBM3 in periampullary and pancreatic adenocarcinoma. In contrast with previous studies on e.g. epithelial ovarian cancer [8], high mRNA levels of RBM3 were found to signify a significantly shorter survival. Moreover, the finding of nuclear RBM3 protein expression being significantly associated with less favorable clinicopathological characteristics and an independent predictor of poor prognosis for patients not receiving adjuvant chemotherapy is in contrast to the vast majority of previous studies, wherein it has been demonstrated to be an independent marker of a favorable clinical outcome [7, 9–13].

However, the strong predictive value of high RBM3 expression with regard to adjuvant treatment is in line with previous findings of RBM3 being predictive of response to platinum-based chemotherapy in epithelial ovarian cancer and colorectal cancer [8, 15]. In addition, the reported significant association between reduced RBM3 expression and treatment failure in patients with non-seminomatous testicular cancer also provides indirect support of a link between RBM3 expression and cisplatin sensitivity [14]. Importantly, the significant interaction found here between RBM3 expression and adjuvant chemotherapy was most evident in PB-type tumors, which are considered to have the poorest clinical outcome, and there are currently no clinical tools for determining which patients will actually benefit from adjuvant treatment and not merely suffer from the adverse side effects resulting in a reduced quality of life. Along this line, the observation that the expression of RBM3 was significantly higher in lymph node metastases compared to primary tumors, in particular in PB-type tumors, is highly relevant, as it is the metastatic tumor component that will have the greatest impact on clinical outcome. It also suggests that biomarker analysis of the primary tumor in resected specimens should be sufficient in the case of RBM3.

The findings of high RBM3 mRNA expression levels being associated with a significantly impaired survival in the TCGA dataset and the negative prognostic impact of RBM3 protein expression in patients not receiving adjuvant treatment, invite to further mechanistic investigations. One can reason that the association between RBM3 and more aggressive clinicopathological characteristics is reflected phenotypically by more proliferating tumors, hence being more responsive to chemotherapy. Supporting this notion, RBM3 is known to be up-regulated in non-malignant proliferating cells [19] and cancer [10, 12, 13, 18]. However, studies on e.g. malignant melanoma and upper gastrointestinal adenocarcinoma have failed to demonstrate associations between RBM3 expression and the proliferation marker Ki67 [13, 20]. Furthermore, the patients with high tumor-specific RBM3 expression who did not receive chemotherapy may not have been fit enough to be given chemotherapy, thus having a worse prognosis regardless. Unfortunately, data on oncologic performance status was not available, which would have provided more insight to the treatment selection and eligibility in this cohort. This is a limitation of the study and should be included in subsequent studies on adjuvant therapy in periampullary cancer patients. Despite this, the only parameters differing significantly between adjuvant treated and non-treated patients with PB-type tumors in the herein investigated cohort was tumor origin and year of surgery, not any established prognostic patient or tumor characteristics [21], which would indeed support a beneficial predictive value of RBM3 expression in gemcitabine-treated patients.

RBM3 was also investigated in a functional setting *in vitro*. Suppression of RBM3 led to reduced sensitivity of MIAPaCa-2 pancreatic cancer cells to chemotherapeutic drugs. This is in line with a previous study on ovarian cancer [8], wherein RBM3 levels were found to be significantly higher in parental A2780 ovarian cancer cells compared to their cisplatin-resistant derivative, and reduced cisplatin-sensitivity upon RBM3 knockdown in the former. Herein, the cell line with the highest RBM3-expression was already completely chemo-resistant. This implicates the existence of other, RBM3-independent, mechanisms involved in sensitivity or resistance to chemotherapy in pancreatic cancer, that may become of importance in some tumors with high RBM3 expression that do not respond to chemotherapy. Furthermore, silencing of RBM3 led to reduced cancer cell migration and invasion, which reflects the findings of high RBM3 expression being associated with unfavorable clinicopathological characteristics, in particular in PB-type tumors, displaying the highest levels of RBM3 expression.

The mechanistic basis for the association between RBM3 expression and a more aggressive phenotype of pancreatic cancer needs to be further elucidated. In colorectal cancer, RBM3 has previously been shown to stabilize the mRNA levels of COX-2 and IL-8 [18]. Both of these proteins have been implicated in pancreatic cancer, with overexpression being associated with angiogenesis, inflammation and invasion [22–24]. In this study, however, silencing of RBM3 did not influence levels of neither COX-2 nor IL-8, and there was no correlation between baseline levels of RBM3 and COX-2 or IL-8. In other settings, RBM3 has been shown to promote cell cycle progression [18, 25], and this might, in cancerous cells, be translated to a facilitated action of chemotherapeutic agents e.g. the incorporation of the nucleoside analogs gemcitabine or 5-FU into DNA. Further studies are required to find other candidate targets and downstream effects of RBM3.

In summary, this study provides a first description of the expression and prognostic significance of RBM3 in periampullary adenocarcinoma, including pancreatic cancer. The results demonstrate high expression of RBM3 in the primary tumor to be a negative prognostic factor but also a strong significant predictor of improved response to adjuvant chemotherapy, in particular gemcitabine. These findings were corroborated by *in vitro* investigations, wherein suppression of RBM3 led to reduced cancer cell invasion and enhanced resistance to chemotherapy. Thus, RBM3 is a promising biomarker candidate for improved treatment stratification of patients with periampullary and pancreatic cancer, and merits further study, in the clinical setting as well as in a functional context.

## MATERIALS AND METHODS

### Restrospective cohort

The main study cohort consists of a retrospective consecutive series of 175 patients surgically treated with pancreaticoduodenectomy for primary periampullary adenocarcinoma at Skåne University Hospital, Malmö and Lund, Sweden, from January 1st 2001 until December 31st 2011. The cohort has previously been described in detail [21, 26–30]. Haematoxylin and eosin stained slides of tissue samples from all patients were re-evaluated by one pathologist (JEL), blinded to the original report and outcome, with the decision on tumor origin and morphological type being based on several criteria as previously described [26]. Data on survival were gathered from the Swedish National Civil Register. Follow-up started at the date of surgery and ended at death or at December 31st 2013, whichever came first. Information on neoadjuvant and adjuvant treatment and recurrence was obtained from patient records.

The study has been approved by the Ethics Committee of Lund University (ref nr 445/07).

### Tissue microarray construction

Tissue microarrays (TMAs) were constructed using a semi-automated arraying device (TMArrayer, Pathology Devices, Westminister, MD, USA). A standard set of three tissue cores (1mm) were obtained from each of the 175 primary tumors and from 105 paired lymph node metastases, whereby one to three lymph node metastases were sampled in each case. Benign-appearing pancreatic tissue was also sampled from 50 patients, using a standard set of two 1 mm tissue cores. To avoid sampling of distant islets of pancreatic cancer, pancreatic parenchyma was only sampled from specimens with duodenal or ampullary cancer, where all pancreatic parenchyma had a normal microscopic appearance. In all sampled specimens there were at least 20 mm between the sampled area and the major lesion.

### Immunohistochemistry and staining evaluation

For immunohistochemical analysis of RBM3 expression, 4 μm TMA-sections were automatically pre-treated using the PT Link system and then stained in an Autostainer Plus (DAKO, Glostrup, Denmark) with the mouse monoclonal anti-RBM3 antibody AMAb90655 (Atlas Antibodies AB, Stockholm, Sweden) diluted 1:100. The specificity of the antibody has been validated previously [8, 9]. The immunohistochemical staining was evaluated by two independent observers who were blinded to clinical and outcome data (LBD and KJ). Scoring differences were discussed to reach consensus. For assessment of nuclear RBM3 expression, the estimated fraction, 0.0–1.0 (1 = 100%), of cells with nuclear RBM3 expression was recorded for each core, as well as the predominant nuclear intensity as 0 (negative), 1 (weak), 2 (moderate) and 3 (strong). Fraction and intensity for each core was multiplied and a mean value of the cores was then calculated and used in the statistical analyses. Cytoplasmic staining was not as evident, and therefore recorded as 0 (absent) or 1 (present). The nuclear RBM3 expression was dichotomized into high and low categories at the median (1.77) for survival analyses (low RBM3 expression ≤ 1.77, high RBM3 expression > 1.77).

### Survival analysis using TCGA samples

Samples from patients with pancreatic adenocarcinoma were collected from The Cancer Genome Atlas (TCGA) project from the Genomic Data Commons (GDC). The FPKM (fragments per kilobase of exon per million mapped reads) values were retrieved and the average FPKM value was used for all individual samples for each tissue to estimate gene expression levels. A cut-off value of 1 FPKM was used as a detection limit across all tissues.

The patients were classified into two groups and their prognoses examined based on the FPKM values. Genes with low expression were excluded i.e. those with a median expression among samples less than one during the analysis. The prognosis of each group of patients was examined by Kaplan-Meier survival estimates. To choose the best FPKM cut-offs for most significant grouping of the patients, FPKM values from the 20th to 80th percentiles were used and significant differences in the survival outcomes of the groups were examined. No clinical information regarding chemotherapy was available for these patients.

### Cell culture

Human pancreatic cancer cell lines BxPC-3, PANC-1 and MIAPaCa-2 and human fetal foreskin fibroblasts (HFFF2) as well as chemotherapeutic drugs were purchased from Sigma-Aldrich and growth medium, fetal bovine serum (FBS) and antibiotics from Nordic Biolabs. The cells were maintained in RPMI1640 or DMEM medium supplemented with 10% FBS and antibiotics (100 U/ml penicillin and 100 μg/ml streptomycin) in a humified 5% CO_2_ atmosphere at 37°C. All *in vitro* reagents were purchased from ThermoFisher Scientific unless stated otherwise.

### siRNA transfection

For siRNA transfection, pancreatic cancer cells were seeded in T-25 flasks (4–7 × 10^5^ cells) and incubated 72h in 37°C. The cells were then washed twice with phosphate buffered saline (PBS) and received growth medium without FBS, together with lipofectamine 2000 and negative control or anti-RBM3 (s11858 + s11860) siRNA in OptiMEM to a final siRNA concentration of 50 nM. The transfection was stopped after 4.5h, medium changed to full growth medium and the cells were left to recover overnight. The following day, cells were harvested and spun down to pellets. The pellets were either fixated, dehydrated and embedded in paraffin for immunohistochemistry or resuspended in RLT buffer (QIAGEN) and stored in -20°C for qPCR.

### Cell viability assay

Following siRNA transfection and 4h incubation with regular growth medium, cells were harvested and reseeded in 96-well plates (2.5 × 10^4^ cells/well). The following day, cells were treated with gemcitabine (0–10 μM), oxaliplatin (0–100 μM) or 5-FU (0–750 μM) and incubated for 72h in regular growth medium. WST-1 was then added to the wells and the plates were read at 450 nm after 1h, with 620 nm used as reference.

### Transwell migration

Cells were moved to inserts (1.5 × 10^5^ cells, 8 μm pores) after siRNA transfection and 4h recovery in regular growth medium. The following morning, after 14h incubation, cells were fixated in 1% formaldehyde and stained with 0.5% crystal violet. Non-migrated cells were removed with cotton swabs. Migration was visualized under light microscope and images taken at 10X magnification with cellSens software.

### Organotypic assay

The 3D organotypic model was set up 24h after siRNA transfection and according to Moutasim et al and Froeling et al [31, 32]. Inserts (3 μm pores) in a 24-wells plate were coated with collagen prior to making the gel (7 parts Collagen I, 1 part DMEM 10X, 1 part FBS, 1 part regular DMEM growth medium). The gel was made from 3.5 parts Collagen I, 3.5 parts Matrigel, 1 part DMEM 10X, 1 part FBS and 1 part cell suspension and added to each collagen-coated insert. The cell suspension contained 2.5 × 10^4^ HFFF2 cells and the gels were left to incubate 1h in 37C. Following gel polymerization, the cancer cells (5 × 10^4^) were harvested and mixed with HFFF2 (2.5 × 10^4^) and seeded on top of the gels. Medium was added to the wells and the model was incubated overnight in 37C. The following day, the medium was removed from the wells and inserts and medium was added to the well up to the bottom of the insert to create an air-liquid interface. The medium was changed every 2–3 days. After 7 days incubation, the gels were fixated in 4% paraformaldehyde and paraffin embedded. The gels sections (3 μm thick) were stained with hematoxylin and eosin for visualization of cancer cell invasion. Representative pictures were taken with cellSens dimension software at 20X magnification.

### Immunocytochemistry

TMAs were constructed from the paraffin-embedded cell pellets in the same manner as the tissue samples, as was the subsequent staining of the cells.

### qPCR

The cell sample were thawed and spun down to remove cell debris. RNA purification was performed using QIAcube with RNeasy mini kit (QIAGEN). Prior to qPCR, cDNA reverse transcription was performed using the High-capacity cDNA reverse transcription kit and total cDNA concentration was determined using Qubit with the DNA HS kit. 10 ng per reaction of each sample was used to run qPCR with RBM3, COX-2 or IL-8 TaqMan gene expression assay (Assay ID Hs00943160_g1, Hs00153133_m1 and Hs00174103_m1, respectively), with sample run in triplicates. GAPDH was used as endogenous control (Assay ID Hs03929097_g1).

### Statistical analysis

Non-parametric Wilcoxon-Rank, Mann-Whitney U and Kruskal-Wallis tests were applied for analyses of differences in the distribution of RBM3 expression in primary tumors and lymph node metastases and according to clinicopathological characteristics. Two patients with PB-type adenocarcinoma received neoadjuvant chemotherapy and were excluded from the statistical analyses. In addition, three patients were excluded from the survival analyses, two with I-type adenocarcinomas on the basis of death due to complications after surgical treatment, and one with PB-type adenocarcinoma on the basis of emigration. Kaplan Meier and the log rank test were applied to estimate differences in 5-year overall survival (OS) and RFS in strata according to high and low expression of RBM3. Recurrence-free survival (RFS) was defined as the time from the date of surgery to the date of locoregional or distant recurrence. Hazard ratios (HR) for death and recurrence within 5 years were calculated by Cox regression proportional hazard’s modeling in both unadjusted analysis and in a multivariable model adjusted for age, sex, T-stage, N-stage, differentiation grade, lymphatic invasion, vascular invasion, perineural invasion, infiltration in peripancreatic fat, resection margins, tumor location and adjuvant chemotherapy. For estimates of interactions between treatment and RBM3, the following interaction variables were constructed: any adjuvant treatment (+/−) × RBM3 (+/−), and gemcitabine-based treatment (+/−) × RBM3 (+/−). All statistical analyses were performed using IBM SPSS Statistics version 22.0 (SPSS Inc., Chicago, IL, USA). For evaluation of the proliferation data, non-linear regression with normalized values (relative to control) was performed using Graphpad prism software. Differences in relative mRNA levels were calculated by student’s T test. All statistical tests were two-sided and a *p*-value of 0.05 was considered to be statistically significant.

## SUPPLEMENTARY MATERIALS FIGURE AND TABLES







## Abbreviations

CI: confidence interval
COX-2: cyclooxygenase 2
HR: hazard ratio
IL-8: interleukin 8
OS: overall survival
RFS: recurrence free survival
TCGA: the cancer genome atlas
TMA: tissue micro array
VEGF: vascular endothelial growth factor

## References

[1] Neoptolemos JP, Moore MJ, Cox TF, Valle JW, Palmer DH, McDonald AC, Carter R, Tebbutt NC, Dervenis C, Smith D, Glimelius B, Charnley RM, Lacaine F (2012). Effect of adjuvant chemotherapy with fluorouracil plus folinic acid or gemcitabine vs observation on survival in patients with resected periampullary adenocarcinoma: the ESPAC-3 periampullary cancer randomized trial. JAMA.

[2] Pancreatric Section, British Society of Gastroenterology; Pancreatic Society of Great Britain and Ireland; Association of Upper Gastrointestinal Surgeons of Great Britain and Ireland; Royal College of Pathologists; Special Interest Group for Gastro-Intestinal Radiology (2005). Guidelines for the management of patients with pancreatic cancer periampullary and ampullary carcinomas. Gut.

[3] Westgaard A, Tafjord S, Farstad IN, Cvancarova M, Eide TJ, Mathisen O, Clausen OP, Gladhaug IP (2008). Pancreatobiliary versus intestinal histologic type of differentiation is an independent prognostic factor in resected periampullary adenocarcinoma. BMC Cancer.

[4] Herreros-Villanueva M, Hijona E, Cosme A, Bujanda L (2012). Adjuvant and neoadjuvant treatment in pancreatic cancer. World J Gastroenterol.

[5] Romiti A, Barucca V, Zullo A, Sarcina I, Di Rocco R, D’Antonio C, Latorre M, Marchetti P (2012). Tumors of ampulla of Vater: A case series and review of chemotherapy options. World J Gastrointest Oncol.

[6] Siegel R, Ma J, Zou Z, Jemal A (2014). Cancer statistics, 2014. CA Cancer J Clin.

[7] Boman K, Segersten U, Ahlgren G, Eberhard J, Uhlen M, Jirstrom K, Malmstrom PU (2013). Decreased expression of RNA-binding motif protein 3 correlates with tumour progression and poor prognosis in urothelial bladder cancer. BMC Urol.

[8] Ehlen A, Brennan DJ, Nodin B, O’Connor DP, Eberhard J, Alvarado-Kristensson M, Jeffrey IB, Manjer J, Brandstedt J, Uhlen M, Ponten F, Jirstrom K (2010). Expression of the RNA-binding protein RBM3 is associated with a favourable prognosis and cisplatin sensitivity in epithelial ovarian cancer. J Transl Med.

[9] Hjelm B, Brennan DJ, Zendehrokh N, Eberhard J, Nodin B, Gaber A, Ponten F, Johannesson H, Smaragdi K, Frantz C, Hober S, Johnson LB, Pahlman S (2011). High nuclear RBM3 expression is associated with an improved prognosis in colorectal cancer. Proteomics Clin Appl.

[10] Jogi A, Brennan DJ, Ryden L, Magnusson K, Ferno M, Stal O, Borgquist S, Uhlen M, Landberg G, Pahlman S, Ponten F, Jirstrom K (2009). Nuclear expression of the RNA-binding protein RBM3 is associated with an improved clinical outcome in breast cancer. Mod Pathol.

[11] Jonsson L, Bergman J, Nodin B, Manjer J, Ponten F, Uhlen M, Jirstrom K (2011). Low RBM3 protein expression correlates with tumour progression and poor prognosis in malignant melanoma: an analysis of 215 cases from the Malmo Diet and Cancer Study. J Transl Med.

[12] Jonsson L, Gaber A, Ulmert D, Uhlen M, Bjartell A, Jirstrom K (2011). High RBM3 expression in prostate cancer independently predicts a reduced risk of biochemical recurrence and disease progression. Diagn Pathol.

[13] Jonsson L, Hedner C, Gaber A, Korkocic D, Nodin B, Uhlen M, Eberhard J, Jirstrom K (2014). High expression of RNA-binding motif protein 3 in esophageal and gastric adenocarcinoma correlates with intestinal metaplasia-associated tumours and independently predicts a reduced risk of recurrence and death. Biomark Res.

[14] Olofsson SE, Nodin B, Gaber A, Eberhard J, Uhlen M, Jirstrom K, Jerkeman M (2015). Low RBM3 protein expression correlates with clinical stage, prognostic classification and increased risk of treatment failure in testicular non-seminomatous germ cell cancer. PLoS One.

[15] Siesing C, Sorbye H, Dragomir A, Pfeiffer P, Qvortrup C, Ponten F, Jirstrom K, Glimelius B, Eberhard J (2017). High RBM3 expression is associated with an improved survival and oxaliplatin response in patients with metastatic colorectal cancer. PLoS One.

[16] Ehlen A, Nodin B, Rexhepaj E, Brandstedt J, Uhlen M, Alvarado-Kristensson M, Ponten F, Brennan DJ, Jirstrom K (2011). RBM3-regulated genes promote DNA integrity and affect clinical outcome in epithelial ovarian cancer. Transl Oncol.

[17] Zeng Y, Wodzenski D, Gao D, Shiraishi T, Terada N, Li Y, Vander Griend DJ, Luo J, Kong C, Getzenberg RH, Kulkarni P (2013). Stress-response protein RBM3 attenuates the stem-like properties of prostate cancer cells by interfering with CD44 variant splicing. Cancer Res.

[18] Sureban SM, Ramalingam S, Natarajan G, May R, Subramaniam D, Bishnupuri KS, Morrison AR, Dieckgraefe BK, Brackett DJ, Postier RG, Houchen CW, Anant S (2008). Translation regulatory factor RBM3 is a proto-oncogene that prevents mitotic catastrophe. Oncogene.

[19] Wellmann S, Truss M, Bruder E, Tornillo L, Zelmer A, Seeger K, Buhrer C (2010). The RNA-binding protein RBM3 is required for cell proliferation and protects against serum deprivation-induced cell death. Pediatr Res.

[20] Nodin B, Fridberg M, Jonsson L, Bergman J, Uhlen M, Jirstrom K (2012). High MCM3 expression is an independent biomarker of poor prognosis and correlates with reduced RBM3 expression in a prospective cohort of malignant melanoma. Diagn Pathol.

[21] Elebro J, Heby M, Gaber A, Nodin B, Jonsson L, Fristedt R, Uhlen M, Jirstrom K, Eberhard J (2014). Prognostic and treatment predictive significance of SATB1 and SATB2 expression in pancreatic and periampullary adenocarcinoma. J Transl Med.

[22] Chu J, Lloyd FL, Trifan OC, Knapp B, Rizzo MT (2003). Potential involvement of the cyclooxygenase-2 pathway in the regulation of tumor-associated angiogenesis and growth in pancreatic cancer. Mol Cancer Ther.

[23] Kuwada Y, Sasaki T, Morinaka K, Kitadai Y, Mukaida N, Chayama K (2003). Potential involvement of IL-8 and its receptors in the invasiveness of pancreatic cancer cells. Int J Oncol.

[24] Li M, Zhang Y, Feurino LW, Wang H, Fisher WE, Brunicardi FC, Chen C, Yao Q (2008). Interleukin-8 increases vascular endothelial growth factor and neuropilin expression and stimulates ERK activation in human pancreatic cancer. Cancer Sci.

[25] Matsuda A, Ogawa M, Yanai H, Naka D, Goto A, Ao T, Tanno Y, Takeda K, Watanabe Y, Honda K, Taniguchi T (2011). Generation of mice deficient in RNA-binding motif protein 3 (RBM3) and characterization of its role in innate immune responses and cell growth. Biochem Biophys Res Commun.

[26] Elebro J, Jirstrom K (2014). Use of a standardized diagnostic approach improves the prognostic information of histopathologic factors in pancreatic and periampullary adenocarcinoma. Diagn Pathol.

[27] Lundgren S, Warfvinge CF, Elebro J, Heby M, Nodin B, Krzyzanowska A, Bjartell A, Leandersson K, Eberhard J, Jirstrom K (2016). The Prognostic Impact of NK/NKT Cell Density in Periampullary Adenocarcinoma Differs by Morphological Type and Adjuvant Treatment. PLoS One.

[28] Fristedt R, Elebro J, Gaber A, Jonsson L, Heby M, Yudina Y, Nodin B, Uhlen M, Eberhard J, Jirstrom K (2014). Reduced expression of the polymeric immunoglobulin receptor in pancreatic and periampullary adenocarcinoma signifies tumour progression and poor prognosis. PLoS One.

[29] Elebro J, Heby M, Warfvinge CF, Nodin B, Eberhard J, Jirstrom K (2016). Expression and Prognostic Significance of Human Epidermal Growth Factor Receptors 1, 2 and 3 in Periampullary Adenocarcinoma. PLoS One.

[30] Elebro J, Ben Dror L, Heby M, Nodin B, Jirstrom K, Eberhard J (2016). Prognostic effect of hENT1, dCK and HuR expression by morphological type in periampullary adenocarcinoma, including pancreatic cancer. Acta Oncol.

[31] Moutasim KA, Nystrom ML, Thomas GJ (2011). Cell migration and invasion assays. Methods Mol Biol.

[32] Froeling FE, Marshall JF, Kocher HM (2010). Pancreatic cancer organotypic cultures. J Biotechnol.

