# Supplementary material for: Translational study reveals a two-faced role of RBM3 in pancreatic cancer and suggests its potential value as a biomarker for improved patient stratification
Source: Oncotarget. 2017 Dec 15;9(5):6188–200. doi: 10.18632/oncotarget.23486 (PMC5814204; doi:10.18632/oncotarget.23486)
Supplement: Supplementary file 1 [file oncotarget-09-6188-s001.pdf]

Translational study reveals a two-faced role of RBM3 in pancreatic cancer and suggests its potential value as a biomarker for improved patient stratification

SUPPLEMENTARY MATERIALS

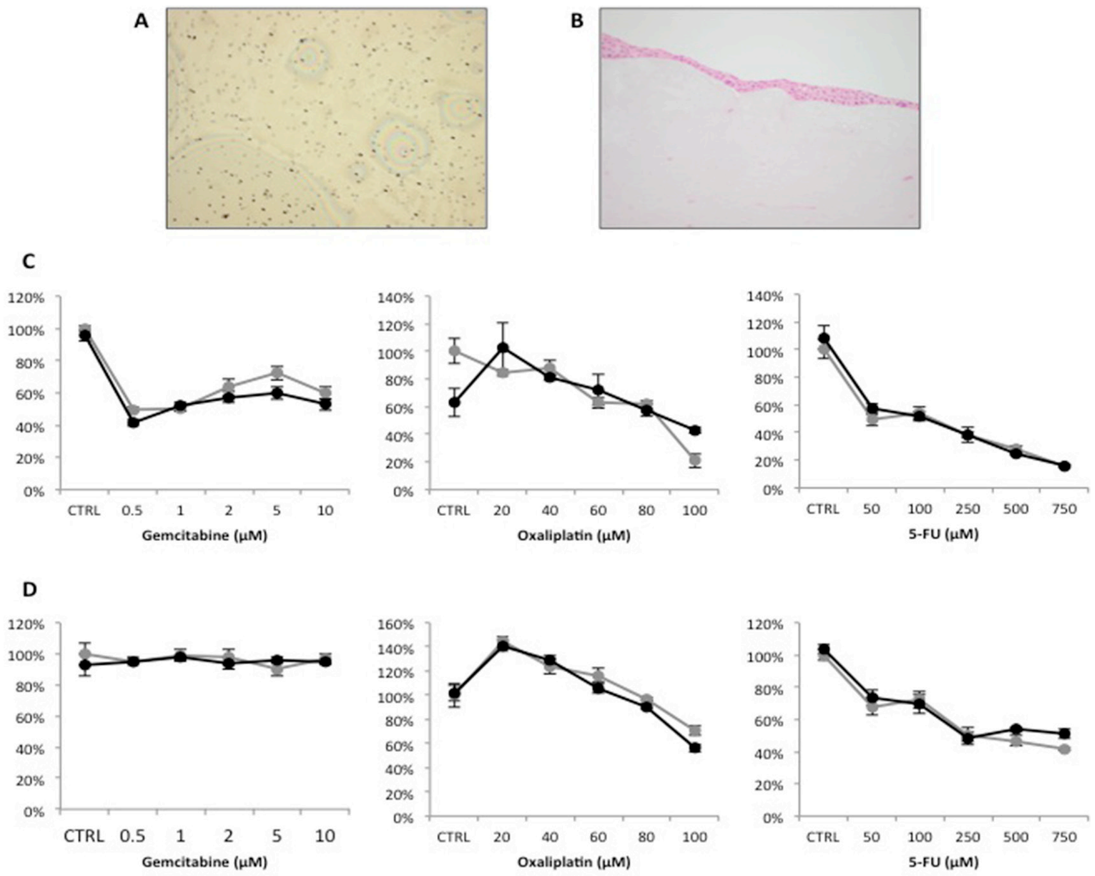

Supplementary Figure 1: Migration and chemotherapy response of pancreatic cancer cells. (**A**) Transwell migration of non-transfected MIAPaCa-2 cells after 14 h incubation. No cells migrated across the membrane, and transfected cells displayed the same results. (**B**) Organotypic gel section after 7 days incubation and stained with hematoxylin and eosin. Non-transfected BxPC-3 cells display no invasion and a clear border between the tumor epithelium on top and the gel. Cells transfected with RBM3 generated the same results. (**C–D**). Graphs represent response to chemotherapy for transfected cells (black lines) and control (grey lines). BxPC-3 cells (C) and PANC-1 cells (D) were treated with indicated doses of gemcitabine, oxaliplatin or 5-FU for 72 h.

Supplementary Table 1: Unadjusted and adjusted hazard ratios for death within 5 years in the entire cohort, intestinal type and pancreatobiliary type tumors. See Supplementary_Table_1

Supplementary Table 2: Unadjusted and adjusted hazard ratios for recurrence in the entire cohort, intestinal type and pancreatobiliary type tumors. See Supplementary_Table_2
