# Supplementary material for: Translational study reveals a two-faced role of RBM3 in pancreatic cancer and suggests its potential value as a biomarker for improved patient stratification
Source: Oncotarget. 2017 Dec 15;9(5):6188–200. doi: 10.18632/oncotarget.23486 (PMC5814204; doi:10.18632/oncotarget.23486)
Supplement: Supplementary file 2 [file oncotarget-09-6188-s002.docx]

**Supplementary Table 1: Unadjusted and adjusted hazard ratios for death within 5 years in the entire cohort, intestinal type and pancreatobiliary type tumors**

|  | **Entire cohort** | | | **Intestinal type** | | | **Pancreatobiliary type** | | |
| --- | --- | --- | --- | --- | --- | --- | --- | --- | --- |
|  |  | Unadjusted | Adjusted |  | Unadjusted | Adjusted |  | Unadjusted | Adjusted |
|  | n(events) | HR(95%CI) | HR(95%CI) | n(events) | HR(95%CI) | HR(95%CI) | n(events) | HR(95%CI) | HR(95%CI) |
| **Age** |  |  |  |  |  |  |  |  |  |
| Continuous | 166 (111) | 1.00 (0.99-1.03) | 1.03 (1.01-1.06) | 61 (30) | 1.02 (0.98-1.06) | 1.05 (1.00-1.09) | 105 (81) | 0.99 (0.96-1.02) | 1.01 (0.98-1.05) |
| **Gender** |  |  |  |  |  |  |  |  |  |
| Female | 84 (49) | 1.00 | 1.00 | 34 (13) | 1.00 | 1.00 | 50 (36) | 1.00 | 1.00 |
| Male | 82 (62) | 1.40 (0.96-2.04) | 1.21 (0.81-1.81) | 27 (17) | 1.85 (0.89-3.84) | 1.83 (0.71-4.72) | 55 (45) | 1.20 (0.77-1.86) | 1.24 (0.78-1.99) |
| **Tumor origin** |  |  |  |  |  |  |  |  |  |
| Duodenum | 13 (5) | 1.00 | 1.00 | 13 (5) | 1.00 | 1.00 |  |  |  |
| Ampulla-Intestinal type | 48 (25) | 1.50 (0.57-3.91) | 1.95 (0.74-5.18) | 48 (25) | 1.49 (0.57-3.88) | 2.49 (0.65-9.54) |  |  |  |
| Ampulla-Pancreatobiliary type | 18 (15) | 4.03 (1.46-11.17) | 1.75 (0.58-5.28) |  |  |  | 18 (15) | 1.00 | 1.00 |
| Distal Bile duct | 44 (32) | 2.94 (1.14-7.56) | 2.01 (0.75-5.38) |  |  |  | 44 (32) | 0.74 (0.40-1.37) | 0.98 (0.48-2.02) |
| Pancreas | 43 (34) | 3.62 (1.41-9.30) | 2.38 (0.87-6.49) |  |  |  | 43 (34) | 0.91 (0.50-1.68) | 1.04 (0.52-2.09) |
| **Tumor size** |  |  |  |  |  |  |  |  |  |
| Continuous | 166 (111) | 1.02 (1.00-1.03) | 1.01 (0.98-1.03) | 61 (30) | 1.00 (0.98-1.03) | 1.02 (0.98-1.05) | 105 (81) | 1.03 (1.01-1.05) | 1.02 (0.99-1.04) |
| **T-stage** |  |  |  |  |  |  |  |  |  |
| T1 | 6 (3) | 1.00 | 1.00 | 4 (2) | 1.00 | 1.00 | 2 (1) | 1.00 | 1.00 |
| T2 | 20 (9) | 1.03 (0.28-3.80) | 0.80 (0.20-3.20) | 10 (3) | 0.65 (0.11-3.88) | 0.79 (0.11-5.54) | 10 (6) | 0.27 (0.04-2.04) | 0.89 (0.09-8.64) |
| T3 | 102 (69) | 2.43 (0.76-7.74) | 1.14 (0.32-4.06) | 25 (9) | 0.94 (0.20-4.37) | 1.33 (0.20-8.81) | 77 (60) | 0.38 (0.14-1.00) | 0.93 (0.11-7.88) |
| T4 | 38 (30) | 2.95 (0.90-9.69) | 1.86 (0.44-7.86) | 22 (16) | 2.55 (0.58-11.15) | 2.37 (0.29-19.11) | 16 (14) | 0.78 (0.44-1.40) | 2.47 (0.11-57.28) |
| **N-stage** |  |  |  |  |  |  |  |  |  |
| N0 | 62 (33) | 1.00 | 1.00 | 33 (15) | 1.00 | 1.00 | 29 (18) | 1.00 | 1.00 |
| N1 | 63 (45) | **2.07 (1.31-3.25)** | **1.73 (1.06-2.82)** | 19 (9) | 1.17 (0.51-2.68) | 0.51 (0.19-1.38) | 44 (36) | **2.40 (1.34-4.28)** | **2.88 (1.58-5.25)** |
| N2 | 41 (33) | 2.71 (1.66-4.44) | **1.76 (1.01-3.07)** | 9 (6) | 2.08 (0.80-5.37) | **3.32 (1.06-10.44)** | 32 (27) | **2.57 (1.39-4.75)** | **2.45 (1.29-4.63)** |
| **Differentiation grade** |  |  |  |  |  |  |  |  |  |
| Well-moderate | 68 (35) | 1.00 | 1.00 | 30 (12) | 1.00 | 1.00 | 38 (23) | 1.00 | 1.00 |
| Poor | 98 (76) | **2.46 (1.64-3.68)** | **1.91 (1.25-2.91)** | 31 (18) | 1.98 (0.95-4.11) | 1.78 (0.69-4.58) | 67 (58) | **2.49 (1.52-4.06)** | **2.06 (1.23-3.47)** |
| **Involved margins, status** |  |  |  |  |  |  |  |  |  |
| R0 | 23 (6) | 1.00 | 1.00 | 17 (4) | 1.00 | 1.00 | 6 (2) | 1.00 | 1.00 |
| R1 & Rx | 143 (105) | **3.82 (1.67-8.69)** | 2.31 (0.99-5.42) | 44 (26) | 2.56 (0.89-7.36) | 0.77 (0.22-2.68) | 99 (79) | 3.48 (0.85-14.19) | 2.55 (0.62-10.53) |
| **Lymphatic growth** |  |  |  |  |  |  |  |  |  |
| Absent | 60 (29) | 1.00 | 1.00 | 28 (7) | 1.00 | 1.00 | 32 (22) | 1.00 | 1.00 |
| Present | 106 (82) | **2.20 (1.43-3.36)** | 1.17 (0.71-1.95) | 33 (23) | **3.61 (1.55-8.44)** | **5.81 (2.07-16.31)** | 73 (59) | 1.51 (0.92-2.47) | 0.99 (0.56-1.75) |
| **Vascular growth** |  |  |  |  |  |  |  |  |  |
| Absent | 126 (72) | 1.00 | 1.00 | 56 (25) | 1.00 | 1.00 | 70 (47) | 1.00 | 1.00 |
| Present | 40 (39) | **3.48 (2.33-5.19)** | **3.16 (2.04-4.89)** | 5 (5) | **7.78 (2.74-22.11)** | **4.77 (1.51-15.10)** | 35 (34) | **2.41 (1.55-3.77)** | **2.49 (1.56-3.97)** |
| **Perineural growth** |  |  |  |  |  |  |  |  |  |
| Absent | 64 (31) | 1.00 | 1.00 | 42 (17) | 1.00 | 1.00 | 22 (14) | 1.00 | 1.00 |
| Present | 102 (80) | **2.64 (1.73-4.03)** | 1.06 (0.62-1.84) | 19 (13) | **2.15 (1.04-4.44)** | **3.06 (1.31-7.14)** | 83 (67) | **1.87 (1.04-3.36)** | 0.94 (0.46-1.92) |
| **Growth in peripancreatic fat** |  |  |  |  |  |  |  |  |  |
| Absent | 62 (28) | 1.00 | 1.00 | 40 (14) | 1.00 | 1.00 | 22 (14) | 1.00 | 1.00 |
| Present | 104 (83) | **3.02 (1.94-4.70)** | **2.03 (1.23-3.34)** | 21 (16) | **3.49 (1.68-7.25)** | 0.91 (0.12-7.14) | 83 (67) | 1.80 (1.00-3.24) | 1.21 (0.63-2.32) |
| **Adjuvant treatment** |  |  |  |  |  |  |  |  |  |
| Absent | 91 (62) | 1.00 | 1.00 | 43 (24) | 1.00 | 1.00 | 48 (38) | 1.00 | 1.00 |
| Present | 75 (49) | 1.03 (0.71-1.51) | **0.66 (0.44-0.98)** | 18 (6) | 0.60 (0.25-1.47) | **0.34 (0.13-0.92)** | 57 (43) | 0.91 (0.58-1.40) | 0.66 (0.42-1.04) |
| **RBM3 median** |  |  |  |  |  |  |  |  |  |
| Low | 83 (56) | 1.00 | 1.00 | 37 (20) | 1.00 | 1.00 | 46 (36) | 1.00 | 1.00 |
| High | 83 (55) | 1.15 (0.79-1.67) | 0.95 (0.62-1.45) | 24 (10) | 0.84 (0.39-1.80) | 0.88 (0.35-2.18) | 59 (45) | 1.06 (0.68-1.64) | 0.95 (0.60-1.51) |
