# Supplementary material for: Translational study reveals a two-faced role of RBM3 in pancreatic cancer and suggests its potential value as a biomarker for improved patient stratification
Source: Oncotarget. 2017 Dec 15;9(5):6188–200. doi: 10.18632/oncotarget.23486 (PMC5814204; doi:10.18632/oncotarget.23486)
Supplement: Supplementary file 3 [file oncotarget-09-6188-s003.docx]

**Supplementary table 2. Unadjusted and adjusted hazard ratios for recurrence in the entire cohort, intestinal type and pancreatobiliary type tumors**

|  | **Entire cohort** | | | **Intestinal type** | | | **Pancreatobiliary type** | | |
| --- | --- | --- | --- | --- | --- | --- | --- | --- | --- |
|  |  | Unadjusted | Adjusted |  | Unadjusted | Adjusted |  | Unadjusted | Adjusted |
|  | n(events) | HR(95%CI) | HR(95%CI) | n(events) | HR(95%CI) | HR(95%CI) | n(events) | HR(95%CI) | HR(95%CI) |
| **Age** |  |  |  |  |  |  |  |  |  |
| Continuous | 166 (116) | 1.00 (0.98-1.02) | 1.01 (0.99-1.04) | 61 (29) | 1.00 (0.96-1.03) | **1.08 (1.03-1.14)** | 105 (87) | 0.98 (0.96-1.01) | 1.00 (0.96-1.03) |
| **Gender** |  |  |  |  |  |  |  |  |  |
| Female | 84 (53) | 1.00 | 1.00 | 34 (11) | 1.00 | 1.00 | 50 (42) | 1.00 | 1.00 |
| Male | 82 (63) | 1.41 (0.98-2.04) | 1.03 (0.68-1.57) | 27 (18) | **2.31 (1.08-4.94)** | **3.12 (1.23-7.93)** | 55 (45) | 1.06 (0.69-1.61) | 0.80 (0.50-1.27) |
| **Tumor origin** |  |  |  |  |  |  |  |  |  |
| Duodenum | 13 (4) | 1.00 | 1.00 | 13 (4) | 1.00 | 1.00 |  |  |  |
| Ampulla-Intestinal type | 48 (25) | 2.16 (0.75-6.19) | 2.63 (0.90-7.66) | 48 (25) | 2.18 (0.76-6.27) | 3.76 (0.78-18.13) |  |  |  |
| Ampulla-Pancreatobiliary type | 18 (15) | **4.64 (1.53-14.02)** | 1.89 (0.58-6.13) |  |  |  | 18 (15) | 1.00 | 1.00 |
| Distal Bile duct | 44 (38) | **5.25 (1.87-14.76)** | **3.61 (1.24-10.53)** |  |  |  | 44 (38) | 1.11 (0.61-2.02) | 1.55 (0.80-3.01) |
| Pancreas | 43 (34) | **5.02 (1.78-14.19)** | 2.96 (1.00-8.79) |  |  |  | 43 (34) | 1.06 (0.58-1.96) | 1.03 (0.54-1.96) |
| **Tumor size** |  |  |  |  |  |  |  |  |  |
| Continuous | 166 (116) | 1.02 (1.00-1.03) | 1.00 (0.98-1.02) | 61 (29) | 1.00 (0.98-1.02) | 1.00 (0.95-1.05) | 105 (87) | 1.03 (1.02-1.05) | 1.01 (0.99-1.04) |
| **T-stage** |  |  |  |  |  |  |  |  |  |
| T1 | 6 (2) | 1.00 | 1.00 | 4 (1) | 1.00 | 1.00 | 2 (1) | 1.00 | 1.00 |
| T2 | 20 (9) | 1.59 (0.34-7.37) | 1.07 (0.22-5.14) | 10 (3) | 1.28 (0.13-12.30) | 9.19 (0.74-113.66) | 10 (6) | 1.60 (0.19-13.35) | 0.70 (0.08-6.49) |
| T3 | 102 (75) | **4.41 (1.08-17.99)** | 1.93 (0.44-8.44) | 25 (9) | 1.86 (0.24-14.71) | **19.33 (1.63-228.67)** | 77 (66) | 4.64 (0.64-33.76) | 1.32 (0.16-10.70) |
| T4 | 38 (30) | **4.83 (1.15-20.29)** | 2.68 (0.52-13.84) | 22 (16) | 5.44 (0.72-41.21) | **68.66 (5.09-926.30)** | 16 (14) | 4.30 (0.56-33.12) | 2.36 (0.11-50.52) |
| **N-stage** |  |  |  |  |  |  |  |  |  |
| N0 | 62 (32) | 1.00 | 1.00 | 33 (11) | 1.00 | 1.00 | 29 (21) | 1.00 | 1.00 |
| N1 | 63 (47) | **2.31 (1.47-3.65)** | **1.93 (1.18-3.16)** | 19 (11) | 2.07 (0.90-4.78) | 1.22 (0.43-3.47) | 44 (36) | **2.17 (1.24-3.78)** | **2.06 (1.13-3.77)** |
| N2 | 41 (37) | **3.82 (2.35-6.22)** | **2.42 (1.39-4.19)** | 9 (7) | **4.06 (1.55-10.59)** | **5.41 (1.53-19.07)** | 32 (30) | **3.09 (1.71-5.59)** | **2.21 (1.17-4.19)** |
| **Differentiation grade** |  |  |  |  |  |  |  |  |  |
| Well-moderate | 68 (39) | 1.00 | 1.00 | 30 (11) | 1.00 | 1.00 | 38 (28) | 1.00 | 1.00 |
| Poor | 98 (77) | **2.25 (1.52-3.32)** | **1.76 (1.16-2.67)** | 31 (18) | 2.16 (1.02-4.57) | 1.26 (0.36-4.43) | 67 (59) | **2.35 (1.46-3.78)** | **2.25 (1.34-3.78)** |
| **Involved margins, status** |  |  |  |  |  |  |  |  |  |
| R0 | 23 (7) | 1.00 | 1.00 | 17 (3) | 1.00 | 1.00 | 6 (4) | 1.00 | 1.00 |
| R1 & Rx | 143 (109) | **4.24 (1.97-9.12)** | **2.29 (1.04-5.05)** | 44 (26) | 4.51 (1.36-14.94) | 2.02 (0.54-7.56) | 99 (83) | 2.30 (0.84-6.33) | 2.25 (0.79-6.35) |
| **Lymphatic growth** |  |  |  |  |  |  |  |  |  |
| Absent | 60 (28) | 1.00 | 1.00 | 28 (5) | 1.00 | 1.00 | 32 (23) | 1.00 | 1.00 |
| Present | 106 (88) | **2.76 (1.80-4.25)** | 1.32 (0.80-2.18) | 33 (24) | **6.16 (2.34-16.19)** | **6.89 (1.85-25.72)** | 73 (64) | **1.77 (1.09-2.88)** | 1.09 (0.62-1.92) |
| **Vascular growth** |  |  |  |  |  |  |  |  |  |
| Absent | 126 (79) | 1.00 | 1.00 | 56 (24) | 1.00 | 1.00 | 70 (55) | 1.00 | 1.00 |
| Present | 40 (37) | **3.48 (2.31-5.24)** | **2.57 (1.64-4.03)** | 5 (5) | **8.16 (2.86-23.30)** | **3.39 (1.03-11.19)** | 35 (32) | **2.34 (1.49-3.67)** | **1.99 (1.22-3.25)** |
| **Perineural growth** |  |  |  |  |  |  |  |  |  |
| Absent | 64 (29) | 1.00 | 1.00 | 42 (15) | 1.00 | 1.00 | 22 (14) | 1.00 | 1.00 |
| Present | 102 (87) | **3.48 (2.25-5.37)** | 1.09 (0.64-1.86) | 19 (14) | 2.72 (1.31-5.66) | 1.04 (0.29-3.72) | 83 (73) | **2.92 (1.57-5.44)** | 1.84 (0.96-3.54) |
| **Growth in peripancreatic fat** |  |  |  |  |  |  |  |  |  |
| Absent | 62 (25) | 1.00 | 1.00 | 40 (12) | 1.00 | 1.00 | 22 (13) | 1.00 | 1.00 |
| Present | 104 (91) | **4.28 (2.71-6.79)** | **2.48 (1.45-4.23)** | 21 (17) | 4.74 (2.23-10.10) | 1.07 (0.13-9.00) | 83 (74) | **2.59 (1.42-4.74)** | 1.68 (0.89-3.19) |
| **Adjuvant treatment** |  |  |  |  |  |  |  |  |  |
| Absent | 91 (60) | 1.00 | 1.00 | 43 (21) | 1.00 | 1.00 | 48 (39) | 1.00 | 1.00 |
| Present | 75 (56) | 1.24 (0.86-1.79) | **0.64 (0.43-0.96)** | 18 (80) | 0.87 (0.38-1.96) | **0.29 (0.10-0.83)** | 57 (48) | 1.08 (0.70-1.65) | 0.78 (0.48-1.26) |
| **RBM3 median expression** |  |  |  |  |  |  |  |  |  |
| Low | 83 (54) | 1.00 | 1.00 | 37 (19) | 1.00 | 1.00 | 46 (35) | 1.00 | 1.00 |
| High | 83 (62) | 1.31 (0.91-1.89) | 1.13 (0.75-1.71) | 24 (10) | 0.87 (0.40-1.87) | 2.33 (0.90-6.03) | 59 (52) | 1.28 (0.83-1.97) | 1.10 (0.68-1.80) |
